# Risk Factors for Postoperative Hypothermia in Patients Undergoing Robot-Assisted Gynecological Surgery: A Retrospective Cohort Study

**DOI:** 10.7150/ijms.73225

**Published:** 2022-06-27

**Authors:** Sung-Ae Cho, Seok-Jin Lee, Sieun Yoon, Tae-Yun Sung

**Affiliations:** 1 Department of Anaesthesiology and Pain Medicine, Konyang University Hospital, Myunggok Medical Research Center, Konyang University College of Medicine, Daejeon, Korea; 2 Department of Anaesthesiology and Pain medicine, Konyang University Hospital, Konyang University College of Medicine, Daejeon, Korea

**Keywords:** Hypothermia, Robotic-Assisted Surgery, Gynecological Surgery, Incidence, Risk Factors

## Abstract

Since postoperative hypothermia increases the morbidity and mortality rates of surgery, identifying its risk factors is an important part of perioperative management. Considering the increasing demand for robot-assisted surgery and other characteristics of conventional laparoscopic surgery, identifying the risk factors for hypothermia in robot-assisted surgery is necessary. However, this has not yet been clearly established. This study aimed to identify the risk factors and incidence rate of postoperative hypothermia in patients undergoing robot-assisted gynecological surgery. In total, 516 patients aged ≥ 19 years undergoing robot-assisted gynecological surgery at a single university hospital between January 2018 and November 2020 were retrospectively analyzed. Postoperative hypothermia was defined as 36.0°C or lower body temperature at the end of the surgery, and multivariate logistic regression analysis was performed to identify the risk factors for postoperative hypothermia. Among the 516 patients, the incidence rate of postoperative hypothermia was 28.1% in 145 patients. The independent risk factors for postoperative hypothermia included body mass index ≤ 22.9 kg/m^2^, baseline heart rate ≤ 73 rate/min, baseline body temperature ≤ 36.8°C, use of intraoperative nicardipine, and amount of administered intravenous fluid larger than 800 mL. Therefore, to prevent hypothermia in patients undergoing robot-assisted gynecological surgery, these risk factors must be considered.

## Introduction

As the demand for minimally invasive surgery increases, robot-assisted surgery has become increasingly popular. In particular, the number of robot-assisted gynecological surgeries is increasing because of its small surgical incision, cosmetic advantages, and improved accuracy of movement during surgery 1. However, different from conventional laparoscopy, in robot-assisted surgery, it is difficult to change the patient's position once surgery has started, and a steep Trendelenburg position is required during surgery 1. Thus, there is a possibility that the response to an emergency situation may be delayed, and identifying the risk factors to minimize complications and improve patient outcomes is necessary.

Among the points to be considered during anesthesia in robot-assisted surgery, identifying and preventing the risk factors for hypothermia are important because hypothermia can increase the risk of morbid cardiac outcomes, surgical site infection, coagulopathy, blood loss, and delayed emergence from anesthesia 2, 3. In addition, robot-assisted surgery has the characteristic of laparoscopic surgery. Not only the patients are continuously exposed to low temperatures 4 because of the carbon dioxide gas insufflation that makes the operation possible, but also the part that can be warmed using a warming device is limited because of the nature of gynecological surgery that requires lithotomy position intraoperatively 5.

Although the characteristics of robot-assisted surgery might increase the risk of hypothermia, studies on the risk factors for postoperative hypothermia in patients undergoing robot-assisted gynecological surgery have not yet been conducted, different from various studies related to the risk factors for postoperative hypothermia in several surgeries 6-8. Therefore, our study aimed to identify the incidence rate and risk factors for postoperative hypothermia in patients undergoing robot-assisted gynecological surgery.

## Materials and Methods

### Study design and population

This retrospective observational study was approved by the Institutional Review Board of Konyang University Hospital (KYUH 2021-01-004) and registered with the Clinical Research Information Service (number KCT0005837). Written informed consent was not obtained owing to the retrospective nature of this study. This study followed the Strengthening the Reporting of Observational Studies in Epidemiology reporting guidelines.

Data were retrospectively collected from the medical records of patients aged over 18 years undergoing robot-assisted gynecological surgery under general anesthesia at Konyang University Hospital from January 2018 to November 2020. Emergency surgery; combined operation; insufficient medical records of covariate data, including core temperature; and conversion to other surgical methods, such as laparotomy or laparoscopic surgery, from robot-assisted surgery were excluded.

### Anesthesia

Without any premedication, all patients arrived at the preoperative holding area. The ambient temperatures of the preoperative holding area and post-anesthetic care unit and operating room were maintained at 22-25°C and 21-24°C in our hospital, respectively.

After arriving at the operating room, the patients were placed in a bed with a cotton blanket and monitored by performing electrocardiogram recording and noninvasive blood pressure, pulse oximetry, and baseline body temperature evaluations. The patients' baseline body temperature was measured on the tympanic membrane using an infrared tympanic thermometer (ThermoScan IRT 4020, Braun GmbH, Kronberg, Germany [accurate to ± 0.2°C for patient temperatures in the range 35.5-42°C, ± 0.3°C for patient temperatures < 35.5°C]). Before the temperature measurement, the thermometer was calibrated and validated according to the manufacturer's instructions. Immediately after anesthetic induction using propofol, rocuronium, and remifentanil, an esophageal thermistor probe (L000412, Gonimed Co., South Korea) was inserted and placed where the heart sounds could be heard loudly through the stethoscope. Intraoperative core temperature was continuously measured using this probe and recorded every 15 min from the insertion of the temperature probe to the end of anesthesia.

Before surgical draping, all patients were undressed and placed in lithotomy position. Forced-air warming blanket was placed on the upper body above the xiphoid process, including the patient's arm with upper body cover (Bair Hugger^TM^ Upper-body Cover Model 62200, Heater Model 505; Arizant Healthcare Inc., USA). After the end of surgical draping, active warming with forced-air warming device was started and was maintained by setting the temperature to 38°C during surgery, and it was stopped when the core temperature was over 37.5°C. In addition, the humidified heating circuit was maintained as long as the core temperature did not exceed 37.5°C. Fluid warmer (Buddy^TM^; Belmont Inc., Billerica, MA, USA) was used at the discretion of the anesthesiologist, and the anesthesia was maintained with sevoflurane (1.5-2.5 vol%) and target-controlled infusion of remifentanil (plasma concentration, 2-4 ng/ml) to maintain 25-50 of Patient State Index (SedLine®; Masimo Corp., Irvine, CA, USA) value. Hypotensive agents or vasopressors were administered at the discretion of the anesthesiologist if blood pressure was high or low despite maintenance of an adequate depth of anesthesia (i.e., 25-50 of Patient State Index) with sevoflurane and remifentanil.

### Variables and data collection

Postoperative hypothermia was defined as a core temperature < 36°C at the end of surgery measured using an esophageal thermistor probe. Moreover, it was classified as mild (35-35.9°C), moderate (34-34.9°C), and severe (≤ 34°C) based on its severity 8. The following data were considered as potential risk factors and were collected and analyzed: baseline body temperature and core temperature at the end of surgery, age, sex, weight, height, body mass index (BMI), American Society of Anesthesiologists physical status (ASA PS), comorbidities, smoking history, alcohol abuse, use of fluid warmer during surgery, baseline systolic blood pressure (SBP) and heart rate (HR), use of vasopressors and hypotensive agents during surgery, amount of intravenous fluid administered during surgery, intraoperative transfusion, and duration of surgery 2,3,6-8.

### Statistical analyses

According to core temperature at the end of surgery, patients were classified into the hypothermia (< 36°C) and normothermia (36-38°C) groups.

In the univariate analysis, continuous variables were analyzed using Student's t-test or the Mann-Whitney U test after assessing the data distribution using the Kolmogorov-Smirnov test. Categorical variables were analyzed using the χ^2^ test, χ^2^ test for trends (linear-by-linear association), or Fisher's exact test, where appropriate. In all analyses, a two-sided *P* < 0.05 was considered significant.

Univariate and multivariate logistic regression analyses were performed to identify variables associated with postoperative hypothermia in patients undergoing robot-assisted gynecological surgery. All variables showing *P* < 0.1 between the two groups in the univariate analysis were included in the multivariate logistic regression analysis to determine the independent predictors of postoperative hypothermia. Two logistic regression models were built for the analysis. Quantitative variables were considered continuous in model 1, whereas in model 2, they were categorized as dichotomous variables. To categorize the quantitative variable into dichotomous variables, the Youden index was used to construct receiver operating characteristic (ROC) curves for predicting postoperative hypothermia. The goodness-of-fit of the models was evaluated using the Hosmer-Lemeshow test. The predicted values of the two models were evaluated using ROC curves, and the areas under the curves were compared using the method of Delong *et al.* 9. All statistical analyses were performed using the Statistical Package for the Social Sciences (SPSS) software (SPSS version 27.0 for Windows, SPSS, IBM Corp, Armonk, NY, USA).

## Results

**Figure 1:**
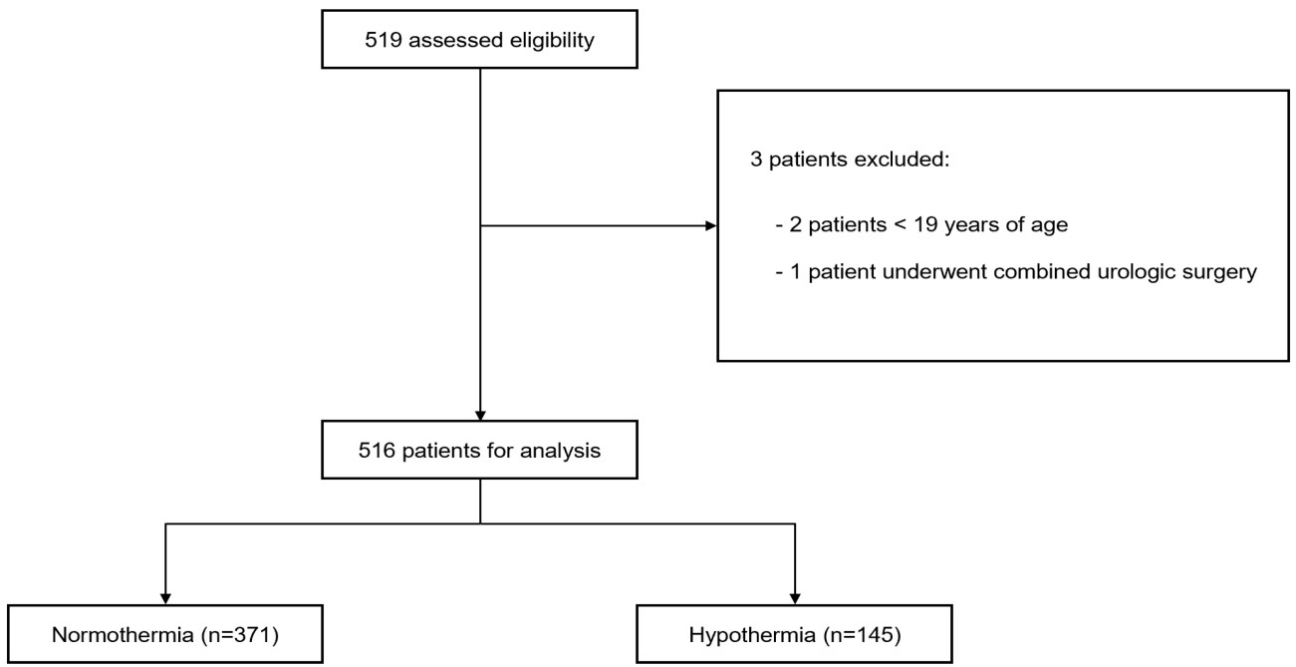
Flowchart of the study.

A total of 519 patients were enrolled in our study according to the inclusion criteria. Of them, three patients were excluded: two because of age < 19 years and one because of combined urological surgery. Therefore, 516 patients were included in this study. Among the analyzed patients, 145 and 371 were classified into the hypothermia and normothermia groups according to the above criteria, respectively (Fig. 1). Among the 516 patients, the incidence rate of postoperative hypothermia was 28.1% in 145 patients. Of these 145 patients, 138 and 7 had mild and moderate hypothermia, respectively, with no severe hypothermia. The demographic and operative data of the two groups are presented in Table 1. The median (interquartile range) baseline body temperatures were 36.8°C (36.5-37.0°C) and 36.9°C (36.7-37.1°C) in the hypothermia and normothermia groups, respectively (*P* < 0.001). The core temperature at the end of surgery was significantly lower compared with baseline body temperature in both groups (*P* < 0.001), and the difference in the core temperature between at the end of surgery and baseline body temperature was significantly larger in the hypothermia group than that in the normothermia group (median [interquartile range], 1.1°C [0.9-1.4°C] vs. 0.4°C [0.2-0.7°C], *P* < 0.001).

According to univariate analysis, BMI, baseline body temperature, baseline HR, use of fluid warmer during surgery, use of intraoperative nicardipine, amount of intravenous fluid, and duration of surgery showed significant differences between the two groups (Table 2). No significant differences were observed in age, ASA PS, patient comorbidities, baseline SBP, use of intraoperative phenylephrine, or intraoperative transfusion.

Multivariate logistic regression analysis was performed with seven variables that showed significant differences in the univariate analysis (Tables 3 and 4). In model 1 of the multivariate logistic regression analysis, lower BMI (odds ratio [OR], 0.860; 95% confidence interval [CI], 0.808-0.916), lower baseline body temperature (OR, 0.302; 95% CI, 0.145-0.628), lower baseline HR (OR, 0.964; 95% CI, 0.944-0.985), use of intraoperative nicardipine (2.585; 95% CI, 1.329-5.026), and larger amounts of intravenous fluid administered (OR, 1.001; 95% CI 1.001-1.002) were independent risk factors for postoperative hypothermia in patients undergoing robot-assisted gynecological surgery (Table 3). In model 2, BMI ≤ 22.9 kg/m^2^ (OR, 3.473; 95% CI, 2.205-5.470), baseline body temperature ≤ 36.8°C (OR, 2.140; 95% CI, 1.380-3.318), baseline HR ≤ 73 rate/min (OR, 2.097; 95% CI, 1.353-3.250), use of fluid warmer (OR, 4.523, 95% CI, 4.5311-15.602), use of intraoperative nicardipine (OR, 2.842; 95% CI, 1.534-5.267), and administered intravenous fluid > 800 mL (OR, 3.958; 95% CI, 2.523-6.208) were independent risk factors for postoperative hypothermia in patient undergoing robot-assisted gynecological surgery (Table 4).

**Figure 2:**
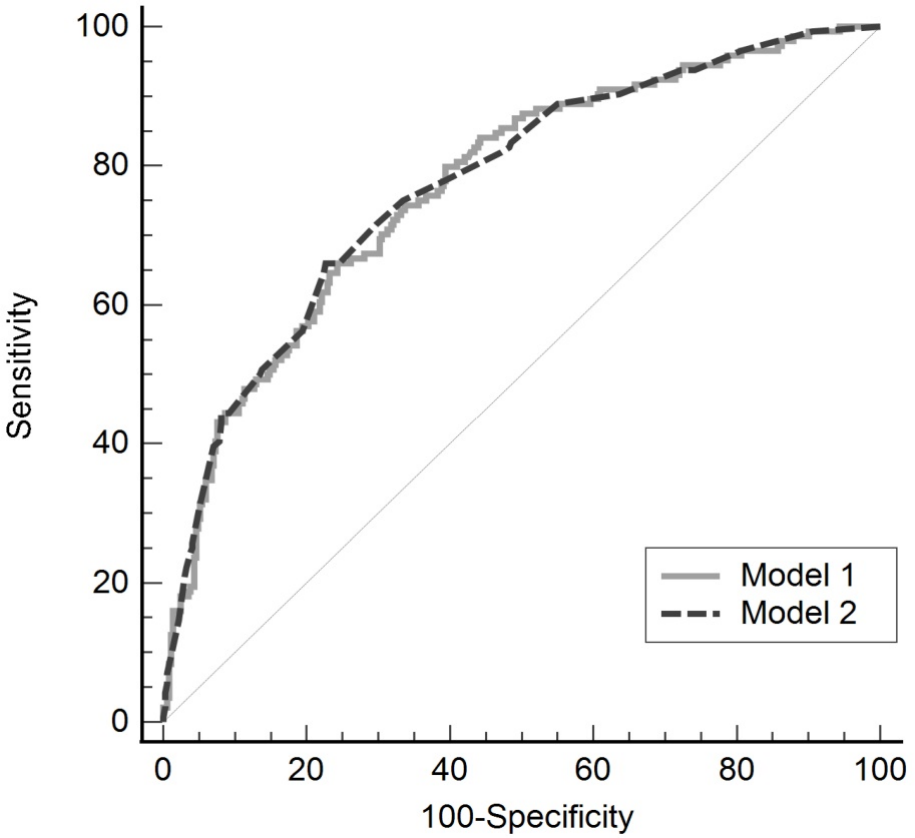
Receiver operating characteristic curve of two multivariate regression models for postoperative hypothermia in robot-assisted gynecological surgery

The validity of the model was confirmed using the Hosmer-Lemeshow test (model 1, *P* = 0.236; model 2, *P* = 0.771). The predictability of these models for postoperative hypothermia in robot-assisted gynecological surgery was evaluated using the ROC curve, and the areas under the curve were 0.771 (standard error, 0.232; 95% CI, 0.732-0.807) and 0.774 (standard error, 0.023; 95% CI, 0.735-0.809), in models 1 and 2 respectively, showing no significant difference between the two models (difference between area, 0.003; *P* = 0.869; Fig. 2).

## Discussion

This study identified the incidence rate of postoperative hypothermia and its risk factors in patients undergoing robot-assisted gynecological surgery. The incidence rate of postoperative hypothermia was 28.1%, and its risk factors included BMI ≤ 22.9 kg/m^2^, baseline HR ≤ 73 rate/min, baseline body temperature ≤ 36.8°C, use of intraoperative fluid warmer and nicardipine, and amount of administered intravenous fluid larger than 800 mL.

Hypothermia in patients receiving general anesthesia occurs due to the impairment of thermoregulation, such as inhibition of vasoconstriction, vasodilation, reduction of metabolic rate, and heat loss due to exposure to cold environment 10. The risk factors for hypothermia identified in this study are related to the causes of hypothermia due to general anesthesia and can also be explained by anesthesia-induced impairments of thermoregulation and characteristics of surgery.

The incidence rate of postoperative hypothermia (28.1%) in this study is comparable to the incidence rate of intraoperative hypothermia (30.6%) in patients undergoing gynecological laparoscopic surgery 11. However, it is lower than the incidence rate of intraoperative hypothermia in obstetric and gynecological surgeries, including open surgery (49.62%) 12. The smaller loss of body via radiation, convection, and evaporation due to smaller surgical site exposure and surgical incision in robot-assisted or laparoscopic surgery than in open surgery may have contributed to this difference in the incidence rate of hypothermia 3. However, in robot-assisted laparoscopic surgery, compared to conventional laparoscopy, once the robot arm is docked and the operation starts, it is difficult to change the patient's position and additional external modality to increase body temperature 1. Therefore, it is necessary to determine the risk factors for hypothermia during robot-assisted surgery.

Lower BMI is a well-known risk factor for perioperative hypothermia in patients receiving general anesthesia 13, 14. Although a higher BMI does not exactly mean a higher fat content, which serves as a buffer for maintaining body temperature during surgery, a lower BMI was confirmed as a risk factor for postoperative hypothermia in patients undergoing robot-assisted gynecological surgery, as in other surgeries, such as laparoscopic, video-assisted thoracoscopic, and orthopedic surgeries 6, 11, 15, 16. Obese patients experience early vasoconstriction when the core temperature decreases to maintain thermal balance due to a higher vasoconstriction threshold, showing less redistribution 17, 18. In addition, the decrease in body temperature and degree of redistribution showed an inverse association with the percentage of fat and body surface area 14.

Additionally, the lower baseline body temperature identified as a risk factor in this study was consistent with that in other studies 2,8,11,15,19. As baseline body temperature refers to the heat content of the periphery and temperature gradient of core-to-peripheral tissue, it is related to the magnitude of redistribution, which is an important cause of anesthesia-induced hypothermia 2,13. With this mechanism, prewarming, which is a technique that adds heat content to the patient exogenously before anesthesia, is considered the most effective method of reducing hypothermia by redistribution, and even short-term prewarming for 10-min was effective in preventing hypothermia in patients undergoing laparoscopic gynecological surgery 5,20. Guidelines for perioperative thermal management recommend prewarming in patients with a pre-anesthesia core temperature of < 36°C 2,3. Our finding that lower baseline body temperature is a risk factor for hypothermia in patients undergoing robot-assisted gynecological surgery supports this recommendation.

In our study, lower baseline HR was a risk factor for hypothermia. A high baseline HR may reflect high levels of preoperative catecholamine secretion, and an increase in plasma catecholamine levels may contribute to the maintenance of thermoregulatory peripheral vasoconstriction 21. However, in this study, baseline SBP did not show intergroup differences; therefore, high levels of preoperative catecholamine alone cannot explain the results. The regulation of heart rate results from the balance between sympathetic and parasympathetic activities, and several factors, such as anxiety, psychological stress, and dehydration, contribute to an increased HR 22. In addition, an increase in HR during anesthesia may increase the risk of hypothermia by causing an increase in cardiac output and promoting redistribution 3. Therefore, further studies on the relevance of HR and hypothermia are required.

Nicardipine is a potent vasodilator commonly used in clinical anesthetic field for the treatment of hypertension 23. In previous studies 24, 25, the use of a vasodilator well before the induction of anesthesia, in which normal body temperature control mechanisms are maintained, resulted in an increase in the heat content of the periphery and a decrease in the temperature gradient between the core and periphery through an increase in perfusion to the periphery, thereby reducing redistribution hypothermia 24. In contrast, intraoperative use of a vasodilator further decreased the core temperature compared with those not used 25. The results of this study are supported by those of a later study. Intraoperative administration of nicardipine in our study for the purpose of lowering blood pressure may have caused a decrease in core temperature by further increasing the core-to-peripheral redistribution of body heat owing to a decrease in systemic vascular resistance 25.

A larger volume of intravenous fluid is also a representative risk factor for hypothermia. It is insufficient to maintain normothermia by administering warmed fluids alone, but conversely, unwarmed fluids administered intravenously cause substantial heat loss via conduction through the surrounding blood 26. The use of 1 L of unwarmed crystalloid reduces mean body temperature by 0.25°C, and the use of > 1 L of unwarmed fluid increases nearly a threefold risk of hypothermia compared with when using < 1 L of unwarmed crystalloid 10,15. Therefore, to prevent hypothermia, the amount of fluid that has not been warmed during surgery should be limited or a fluid warmer should be applied when > 500 mL of fluid is used 10. However, the use of a fluid warmer was identified as a risk factor in model 2 of the multivariate logistic regression analysis in this study, as opposed to studies suggesting that warming fluids prevent hypothermia 26. This is probably because the fluid warmer was used at the discretion of the anesthesiologist in this retrospective study; the fluid warmer would have been applied to patients who have already developed hypothermia or are at high risk of hypothermia during surgery. In addition, a relatively high OR might be expressed considering OR's characteristic wherein only relevance could be confirmed. In the same context, although the amount of blood loss and transfusion are associated with hypothermia 26, in this study, the amount of blood loss could not be accurately assessed due to the nature of the retrospective study, and transfusion would not be identified as a risk factor because the number of patients who received blood transfusion was too small. Furthermore, age, sex, and duration of surgery, which are known risk factors for hypothermia after surgery 6,11,19, were not identified as risk factors in this study probably due to the following characteristics of robot-assisted gynecological surgery. Most patients in our study were middle-aged women, and the constant duration of surgery was observed in the present study due to the limited types of surgery in a single-center study. However, this is the first study to confirm the incidence rate and risk factors for postoperative hypothermia in patients undergoing robot-assisted surgery, and the predictabilities of models 1 and 2 evaluated with ROC curve were good at 77.1% and 77.4%, respectively. Therefore, it is considered to be helpful for future studies related to hypothermia in robot-assisted surgery, which is increasingly required.

Our study has some limitations. First, body temperature before general anesthesia was measured using an infrared tympanic thermometer. The tympanic temperature obtained using an infrared thermometer is the “near-core” temperature and only provides an indirect estimate of the core temperature 2. However, it is easily accessible and widely used in clinical settings as a noninvasive method for measuring body temperature 8,15. Moreover, a recent study demonstrated that an infrared tympanic thermometer has the highest accuracy among other thermometry techniques that can be used for awake patients 3. Second, this study was conducted on patients receiving robot-assisted gynecological surgeries with the same surgical technique by a limited number of surgeons at a single institution. As mentioned above, several known risk factors in previous studies did not become risk factors in our study, probably because of the limited patient population and type of surgery. Therefore, further studies are required to evaluate its external validity. Third, we could not analyze the effect of ambient operating room temperature on postoperative hypothermia owing to the retrospective nature of this study. The effect of ambient operating room temperature is negligible in patients who received intraoperative active forced-air warming, as in our study 27. However, as the part that can be warmed in robot-assisted gynecological surgery is limited to the upper body, it might have affected the postoperative hypothermia.

## Conclusion

In conclusion, the incidence rate of postoperative hypothermia in patients undergoing robot-assisted gynecological surgery was 28.1%. Lower BMI, lower baseline HR, lower baseline body temperature, use of intraoperative nicardipine, and larger amounts of administered intravenous fluid were associated with an increased risk of postoperative hypothermia. While monitoring the intraoperative core temperature and understanding the characteristics of robot-assisted gynecological surgery, anesthetic management to avoid the identified risk factors for postoperative hypothermia in robot-assisted gynecological surgery would be required.

## Author Contributions

All the listed authors were involved in the drafting of the work, approved the final manuscript, and agreed to be accountable for all aspects of this work. Sung-Ae Cho helped in writing the manuscript and analyzing and interpreting data. Seok-Jin Lee helped in writing the manuscript and in the acquisition of data. Sieun Yoon helped in the acquisition, analysis, and interpretation of data. Tae-Yun Sung helped with the conception and design of the study, statistical analysis, and the writing of the manuscript.

## Figures and Tables

**Table 1:** Demographic and operative data in patients with normothermia and hypothermia

Variables	Normothermia (n = 371)	Hypothermia (n = 145)	P
Age, years	46 (40-50)	46 (39-49.5)	0.873
Body mass index, kg/m^2^	24.2 (21.7-27.1)	22.3 (20.8-24.9)	< 0.001
ASA physical status ≥ II	246 (66.3%)	98(67.6%)	0.836
Hypertension	50 (13.5%)	17 (11.7%)	0.594
Diabetes mellitus	23 (6.2%)	5 (3.4%)	0.215
Hypothyroidism	10 (2.7%)	2 (1.4%)	0.524
Hyperthyroidism	5 (1.3%)	2 (1.4%)	> 0.999
Current smokers	18 (4.9%)	10 (6.9%)	0.357
Alcohol abuse	17 (4.6%)	4 (2.8%)	0.460
Baseline body temperature, °C	36.9 (36.7-37.1)	36.8 (36.5-37.0)	< 0.001
Baseline systolic BP, mmHg	130 (117-145)	126 (115.5-147)	0.533
Baseline heart rate, rate/min	75 (70-83)	70 (65-79)	< 0.001
Use of fluid warmer during surgery	5 (1.3%)	8 (5.5%)	0.007
Intraoperative phenylephrine	289 (77.9%)	119 (82.1%)	0.295
Intraoperative nicardipine	33 (8.9%)	31 (21.4%)	< 0.001
Intraoperative transfusion	2 (0.5%)	1 (0.7%)	> 0.999
Intravenous fluid administered, mL	650 (500-900)	900 (600-1450)	< 0.001
Duration of surgery, min	90 (65-130)	105 (65-157.5)	< 0.001

Data are presented as the median (interquartile range), number, or number (%). ASA: American Society of Anesthesiologists; BP: blood pressure; OR: operating room.

**Table 2:** Univariate regression analysis for postoperative hypothermia in patients undergoing robot-assisted gynecological surgery

	Unadjusted odds ratio	95% CI	P
Age, years	1.000	0.980-1.020	0.978
Body mass index, kg/m^2^	0.910	0.864-0.958	< 0.001
ASA physical status ≥ II	1.060	0.704-1.595	0.782
Hypertension	0.853	0.474-1.534	0.595
Diabetes mellitus	0.540	0.201-1.450	0.222
Hypothyroidism	0.505	0.109-2.33	0.381
Hyperthyroidism	1.024	0.196-5.337	0.978
Current smokers	1.453	0.654-3.227	0.359
Alcohol abuse	0.591	0.195-1.786	0.351
Baseline body temperature, °C	0.291	0.153-0.554	< 0.001
Baseline systolic BP, mmHg	0.999	0.990-1.008	0.792
Baseline heart rate, rate/min	0.961	0.942-0.980	< 0.001
Use of fluid warmer during surgery	4.274	1.375-13.291	0.012
Intraoperative phenylephrine	1.299	0.796-2.120	0.296
Intraoperative nicardipine	2.785	1.632-4.752	< 0.001
Intraoperative transfusion	1.281	1.115-14.239	0.840
Intravenous fluid administered, mL	1.001	1.001-1.001	< 0.001
Duration of surgery, min	1.004	1.001-1.007	0.004

CI: confidence interval; ASA: American Society of Anesthesiologists; BP: blood pressure; OR: operating room.

**Table 3:** Multivariate logistic regression analysis: independent risk factors for postoperative hypothermia in patients undergoing robot-assisted gynecological surgery

	β	Adjusted odds ratio	95% CI	P
Body mass index, kg/m^2^	-0.152	0.859	0.807-0.914	< 0.001
Baseline heart rate, rate/min	-0.036	0.965	0.945-0.985	< 0.001
Baseline body temperature, °C	-1.128	0.324	0.157-0.666	0.002
Intraoperative nicardipine	0.943	2.569	1.332-4.954	0.005
Intravenous fluid administered, mL	0.001	1.001	1.001-1.001	< 0.001

In this analysis, quantitative variables were considered continuous variables. CI: confidence interval; OR: operating room.

**Table 4:** Multivariate logistic regression analysis: independent risk factors for postoperative hypothermia in patients undergoing robot-assisted gynecological surgery

	β	Adjusted odds ratio	95% CI	P
Body mass index ≤ 22.9 kg/m^2^	1.245	3.473	2.205-5.470	< 0.001
Baseline heart rate ≤ 73 rate/min	0.740	2.097	1.353-3.250	< 0.001
Baseline body temperature ≤ 36.8°C	0.761	2.140	1.380-3.318	< 0.001
Use of fluid warmer during surgery	1.509	4.523	1.311-15.602	0.017
Intraoperative nicardipine	1.045	2.842	1.534-5.267	< 0.001
Intravenous fluid administered > 800 mL	1.376	3.958	2.523-6.208	< 0.001

In this analysis, quantitative variables were categorized as dichotomous. CI: confidence interval; OR: operating room.

## Abbreviations

ASA PS: American Society of Anesthesiologists physical status
BMI: body mass index
CI: confidence interval
HR: heart rate
OR: odds ratio
ROC: receiver operating characteristic
SBP: systolic blood pressure
SPSS: Statistical Package for the Social Sciences
